# Prescription drug monitoring programs and perioperative opioid prescribing and adverse events

**DOI:** 10.1093/haschl/qxaf218

**Published:** 2025-11-12

**Authors:** Joanne Constantin, Jennifer Waljee, Thuy Nguyen, Amy Bohnert, Usha Nuliyalu, Chad M Brummett, Kao-Ping Chua

**Affiliations:** Susan B. Meister Child Health Evaluation and Research Center, Department of Pediatrics, University of Michigan Medical School, Ann Arbor, MI 48109, United States; Department of Health Policy and Management, School of Public Health, State University of New York (SUNY) Downstate Health Sciences University, Brooklyn, NY 11203, United States; Division of Plastic and Reconstructive Surgery, Indiana University School of Medicine, Indianapolis, IN 46202, United States; Department of Health Management and Policy, University of Michigan School of Public Health, Ann Arbor, MI 48109, United States; Department of Anesthesiology, University of Michigan Medical School, Ann Arbor, MI 48109, United States; Opioid Research Institute, Office of the Vice President for Research, University of Michigan, Ann Arbor, MI 48109, United States; Susan B. Meister Child Health Evaluation and Research Center, Department of Pediatrics, University of Michigan Medical School, Ann Arbor, MI 48109, United States; Department of Anesthesiology, University of Michigan Medical School, Ann Arbor, MI 48109, United States; Opioid Research Institute, Office of the Vice President for Research, University of Michigan, Ann Arbor, MI 48109, United States; Overdose Prevention Engagement Network, University of Michigan Medical School, Ann Arbor, MI 48109, United States; Susan B. Meister Child Health Evaluation and Research Center, Department of Pediatrics, University of Michigan Medical School, Ann Arbor, MI 48109, United States; Department of Health Management and Policy, University of Michigan School of Public Health, Ann Arbor, MI 48109, United States

**Keywords:** prescription drug monitoring programs, health policy, Medicare, surgery, opioid prescribing

## Abstract

**Introduction:**

Most states mandate clinicians to review prescription drug monitoring program (PDMP) databases before prescribing opioids, but the association with perioperative outcomes is unknown.

**Methods:**

We analyzed 2016-2019 Medicare claims for patients ≥65 undergoing surgery in 21 states that enacted PDMP use mandates in 2017-2018 vs states without mandates during 2016-2019. Procedure-level, difference-in-differences models adjusted for patient demographics, prior opioid use, mental health, prior substance use disorders, comorbidities, surgeon specialty, and procedure type. Outcomes included discharge opioid prescriptions, days supplied, total and daily morphine milligram equivalents, high-risk prescribing (≥7-day supply or opioid-benzodiazepine overlap), and 30-day post-discharge adverse events (overdose, emergency department visit, hospitalization, or death).

**Results:**

Among 597 455 procedures for 462 290 patients (mean age 73.3 years; 54.5% female), mandates were not associated with changes in opioid prescribing, high-risk prescribing, or adverse events (difference-in-differences estimate: −0.03% points, 95% CI: −1.0, 0.9). Findings were consistent among patients with prior substance use or in states with stringent mandates.

**Conclusion:**

PDMP use mandates were not associated with changes in perioperative opioid prescribing or post-surgical adverse events, suggesting that policymakers should consider pairing mandates with evidence-based, procedure-specific prescribing guidelines.

Key Points

- Prescription drug monitoring program (PDMP) use mandates were not associated with significant changes in perioperative opioid prescribing or adverse clinical events among Medicare beneficiaries.
- Findings highlight the limited short-term impact of PDMP use mandates on surgical opioid prescribing.
- Policymakers should consider complementary strategies such as prescriber education or payer-based limits to effectively reduce high-risk opioid use in postoperative settings.

## Introduction

In 2024, 54 000 Americans died from an opioid overdose.^1,2^ While illicit opioids are currently the leading driver of opioid-related deaths,^2^ prescription opioids remain a critical point of intervention given their role in initiating opioid misuse and subsequent transition to illicit opioid use.^3-5^ Perioperative care is a common setting for opioid prescribing, where opioids are frequently used to manage acute postoperative pain.^6^ Despite recent decreases in perioperative opioid prescribing, prescribing still often exceeds patient needs,^7^ leading to leftover medications that may be misused or diverted.^8,9^ Additionally, high-risk opioid prescribing patterns after surgery, including opioid-benzodiazepine co-prescription or high daily doses, remain common, further exacerbating the potential for opioid-related harm.^10-13^ These considerations suggest that there are important opportunities to improve the safety and appropriateness of perioperative opioid prescribing.

Prescription drug monitoring programs (PDMPs) are state-regulated electronic databases that provide real-time data on controlled substance prescription dispensing.^14-17^ To date, 40 states have implemented mandates to query these databases before prescribing opioids in at least some circumstances between 2014 and 2019.^18,19^ These “PDMP use mandates” aim to enhance prescribers' ability to identify concerning patterns of controlled substance dispensing, such as “doctor and pharmacy shopping”, a risk factor for opioid overdose mortality.^20-23^ Furthermore, these mandates aim to help mitigate overdose risk by increasing prescribers' awareness of concurrent prescriptions for medications known to exacerbate opioid-related harm, such as benzodiazepines.^11,24-26^ This focus is especially critical for the Medicare population, who face heightened opioid-related risks due to polypharmacy, frailty, and comorbidities. Older adults typically use more than 5 medications concurrently, elevating the risk of adverse interactions in the postoperative period.^27-29^ Simultaneously, substance use disorders, including opioid use disorder, are increasing in this group, affecting nearly 1.7 million beneficiaries annually.^30-32^ Frailty further compounds the risk, contributing to higher mortality and healthcare utilization.^33-35^ Moreover, Medicare beneficiaries are disproportionately affected by prolonged postoperative opioid use, which is associated with falls, hospitalizations, and mortality.^36-38^ These intersecting vulnerabilities highlight the need to understand whether opioid policy interventions, such as PDMP use mandates, can meaningfully improve outcomes for older surgical patients.

Existing research has examined the impact of PDMP use mandates on opioid prescribing across various clinical settings.^24-26,39-45^ Evidence suggests that stringent mandates that require PDMP queries in most or all situations are associated with more substantial reductions in opioid prescribing rates, prescription sizes, and high-risk prescribing compared to less stringent mandates.^41-45^ Surgical settings pose unique challenges; prescribing is episodic, driven by acute pain, and often involves multiple prescribers under one surgeon, leading to variable durations and less standardized monitoring. Surgeons may also underutilize PDMPs, viewing the risk of doctor shopping as minimal in postoperative care, which may yield distinct prescribing patterns compared to other settings. Despite these nuances, evidence on the association between PDMP use mandates and opioid prescribing in surgical settings remains limited.^46-52^ Existing studies have either focused on one state,^47-52^ focused on the pediatric population,^51^ or evaluated mandates implemented prior to 2013.^46^ Moreover, few studies have evaluated the association between PDMP use mandates and high-risk opioid prescribing or adverse events. In this study, we used national Medicare claims data to evaluate our hypothesis that PDMP use mandates improve perioperative opioid prescribing, high-risk prescribing and postoperative adverse events.

## Study data and methods

### Data source

Data on surgical procedures and opioid prescribing and related adverse outcomes were obtained from a 20% sample of Medicare claims from 2016 to 2019.^53,54^ Data on PDMP use mandates came from detailed policy databases from the Prescription Drug Abuse Policy System (PDAPS). Prescription Drug Abuse Policy System is a repository of substance-related policies maintained by the Center for Public Health Law Research at Temple University and funded by the National Institute on Drug Abuse. Legal epidemiologists at PDAPS created a database of state opioid prescribing limits and PDMP use mandates enacted by the end of 2019.^55^ As data were de-identified, the Institutional Review Board of the University of Michigan Medical School exempted this study from human subjects review; informed consent was not required. This manuscript follows the Strengthening the Reporting of Observational Studies in Epidemiology (STROBE) reporting guidelines for cross-sectional studies.^56^

### Study design

The study employed a difference-in-differences design that leveraged state-level variation in the timing of implementation of PDMP use mandates to compare changes in procedure-level outcomes between patients undergoing procedures in treatment and control states (Appendix Table S1). Treatment states included 21 states that enacted PDMP use mandates relevant to perioperative opioid prescribing during 2017-2018. Control states included 10 states that did not enact such PDMP use mandates during 2016-2019. States concurrently enacting PDMP use mandates and opioid prescribing limits,^55^ and those enacting PDMP use mandates prior to 2016, were not included in either the treatment or control group (Appendix Table S2).

### Sample

We defined the index date as the date of discharge for an outpatient procedure or the date of discharge from an inpatient or observation stay in which surgery occurred. Using a previously published crosswalk between procedure codes for major surgical procedures and approximately 1000 procedures,^57,58^ we identified adults 65 years and older undergoing major surgical procedures with an index date between January 1, 2016 and December 31, 2019. We excluded procedures for enrollees younger than 65 and on Medicare for other reasons, those without Medicare Part D coverage, as well as those for patients who lacked continuous medical and pharmacy coverage during the 180 days preceding and 30 days following the index date. Additionally, we excluded procedures with incomplete or implausible opioid dosing data (missing days supplied, days supplied ≤0, days supplied >90, missing quantity, quantity <1) and procedures in which the discharge opioid prescription was for an opioid that lacked a morphine milligram equivalent (MME) conversion factor. To minimize the influence of extreme values, we excluded procedures in which the discharge opioid prescription was in the top or bottom 1% of total MMEs, a standardized measure of prescription size, which excluded 0.5% of procedures.^59,60^ We also excluded procedures for which another surgical procedure occurred within a 180-day look-back or 30-day look-forward period, those associated with a hospital length of stay exceeding 30 days, inpatient procedures in which patients were not discharged home (eg, discharged to a skilled nursing facility or died in the hospital), and procedures with missing covariate data, including age, sex, and race and ethnicity classification. We limited to procedures for patients in treatment and control states. Finally, we excluded surgical procedures in which the service provider was not a surgeon since the analysis focused on PDMP use mandate requirements specific to surgeons.

### Study variables

There were 7 procedure-level outcomes. Surgical opioid prescribing was measured by the following 4 outcomes: an indicator for having a discharge opioid prescription (a dispensed opioid prescription within 3 days of the index date); days supplied in the discharge prescription; total MMEs of the discharge prescription; and daily MME (total MME/days supplied) (Appendix Table S3). High-risk opioid prescribing was measured by the following 2 outcomes: indicator for whether the discharge prescription exceeded a 7-day supply; and indicator for whether the discharge opioid prescription overlapped with a benzodiazepine prescription.^11,61-66^ Adverse events were measured by an indicator for any of the following within 30 days of discharge from surgery: claim with a diagnosis code for opioid overdose, any emergency department (ED) visit, any hospitalization, or death (Appendix Table S4 presents the opioid overdose diagnosis codes that were used). The outcome assessing having a discharge opioid prescription and having an adverse event was calculated for all procedures. The remaining outcomes were only calculated for procedures with a discharge opioid prescription.

### Statistical analysis

Implementation of PDMP use mandates was staggered over the study period. Given that the average treatment effect estimate from a 2-way fixed effects difference-in-differences model could be biased if effects change over time, we employed the approach developed by Callaway and Sant’Anna. In this approach, each cohort of treatment states (based on the month-year of PDMP use mandate adoption) was compared to states that never implemented PDMP use mandates. Cohort-specific effect estimates were aggregated into one treatment effect estimate.^67^ We adjusted for patient demographic characteristics (age, sex, race, and ethnicity), opioid dispensing in the 180 days prior to the index date, surgeon specialty, procedure type, and co-morbidities, including mental health conditions, substance use disorders (including alcohol use disorders), and the Charlson Comorbidity Index. Comorbidities were based on diagnosis codes in the 180 days before the index date through the index date. Race and ethnicity was included as a covariate owing to well-documented disparities in opioid prescribing patterns.^68-71^ We used the variable for self-reported race and ethnicity developed by Research Triangle International^72,73^ (eMethods S1) provides details on the covariates. The Callaway and Sant’Anna inverse probability weighting estimator with stabilized weights was employed, and standard errors were clustered at the state level. To evaluate the parallel trends assumption, we conducted a Callaway and Sant’Anna event study analysis in which the reference month was the month before the implementation of PDMP use mandates.

All statistical analyses were conducted using Stata 18.1/MP (Stata Corp, College Station, TX).^74^ Two-sided hypothesis tests were conducted with α=0.05.

### Subgroup analyses

To explore potential heterogeneity in policy effects, we conducted several subgroup analyses. First, analyses were repeated for patients with prior alcohol or substance use disorders, a risk factor for opioid misuse.^75,76^ Second, analyses were similarly repeated for patients with prior opioid use. Third, analyses were repeated when limiting the treatment group to 5 states with the most stringent PDMP use mandates, defined as mandates that required queries every time a Schedule II opioid was prescribed.

### Limitations

First, due to data limitations, the study could not evaluate the association between PDMP use mandates and patient pain control. However, there is little to no reason to suspect such an association exists given the lack of change in opioid prescribing and given that prior studies have not found evidence that mandates worsen perioperative pain control.^77^ Second, adverse events were only assessed over a short period. However, it is unlikely that using a longer follow-up period would have changed conclusions given the lack of change in opioid prescribing or high-risk opioid prescribing. Third, findings may not generalize to all states or to younger patients. Fourth, as noted below, the average of the pre-intervention coefficients in the event-study analysis was not significant for any outcome, except for the number of days supplied in discharge opioid prescriptions. Thus, caution is warranted for interpreting findings on days supplied. Fifth, despite the study's large sample size, it is possible that the study was underpowered to detect small but potentially clinically meaningful changes in outcomes, such as the occurrence of adverse events.

## Results

### Sample characteristics

Among 4 562 726 procedures initially included, 3 965 271 (86.9%) were excluded, leaving 597 455 procedures for 462 290 patients (Appendix Figure S1). Table 1 shows sample characteristics at the procedure level overall and in the treatment and control groups. Of the 597 455 procedures, 492 523 (82.4%) were in treatment states and 104 932 (17.6%) were in control states. The majority of procedures were for female (325 385; 54.5%) and non-Hispanic White patients (542 680; 90.8%). The mean (SD) patient age at the time of the procedure was 73.3 (6.1) years. Most of the procedures were for patients that did not have prior opioid use in the 180 days preceding the index date (342 169; 57.3%). The most frequent specialty for which opioids were prescribed was orthopedic surgery (236 309; 39.6%). Among all procedures, 59 366 (9.9%) were for patients with a prior alcohol or substance use disorder and 327 234 (54.8%) had a discharge opioid prescription.

**Table 1.** Sample characteristics at the procedure level.

	Total sample *N* = 597 455 (100%)	Treatment *N* = 492 523 (82.4%)	Control *N* = 104 932 (17.6%)
**Mean age (SD)**	73.3 (6.1)	73.3 (6.1)	73.1 (6.1)
**Sex**			
Male	272 070 (45.5%)	224 984 (45.7%)	47 086 (44.9%)
Female	325 385 (54.5%)	267 539 (54.3%)	57 846 (55.1%)
**Race and ethnicity**			
Hispanic, any race	18 396 (3.1%)	17 240 (3.5%)	1156 (1.1%)
Non-Hispanic American Indian/Alaska Native	1343 (0.2%)	945 (0.2%)	398 (0.4%)
Non-Hispanic Asian/Pacific Islander	7233 (1.2%)	6735 (1.4%)	498 (0.5%)
Non-Hispanic Black	24 409 (4.1%)	21 332 (4.3%)	3077 (2.9%)
Non-Hispanic, other race	3394 (0.6%)	3008 (0.6%)	386 (0.4%)
Non-Hispanic White	542 680 (90.8%)	443 263 (90.0%)	99 417 (94.7%)
**Health conditions**			
Prior opioid use			
Yes	255 286 (42.7%)	213 580 (43.4%)	41 706 (39.7%)
No	342 169 (57.3%)	278 943 (56.6%)	63 226 (60.3%)
Any mental health condition			
Yes	165 835 (27.8%)	137 453 (27.9%)	28 382 (27.0%)
No	431 620 (72.2%)	355 070 (72.1%)	76 550 (73.0%)
Alcohol or substance use disorder			
Yes	59 366 (9.9%)	48 625 (9.9%)	10 741 (10.2%)
No	538 089 (90.1%)	443 898 (90.1%)	94 191 (89.8%)
CCI			
>0	430 451 (72.0%)	356 979 (72.5%)	73 472 (70.0%)
0	167 004 (28.0%)	135 544 (27.5%)	31 460 (30.0%)
Surgeon's specialty			
Orthopedic surgery	236 309 (39.6%)	192 635 (39.1%)	43 674 (41.6%)
General surgery	133 173 (22.3%)	110 563 (22.4%)	22 610 (21.5%)
Urology	35 734 (6.0%)	29 630 (6.0%)	6104 (5.8%)
Otolaryngology	33 028 (5.5%)	27 249 (5.5%)	5779 (5.5%)
Neurological surgery	32 869 (5.5%)	26 622 (5.4%)	6247 (6.0%)
Plastic surgery	29 525 (4.9%)	25 192 (5.1%)	4333 (4.1%)
Vascular surgery	27 426 (4.6%)	22 802 (4.6%)	4624 (4.4%)
Other specialty	69 391 (11.6%)	57 830 (11.9%)	11 561 (11.1%)

Source: The 2016-2019 Medicare claims data.

CCI, Charlson Co-Morbidity Index; SD, standard deviation.

Mental health conditions include adjustment disorders, anxiety, mood disorders, suicide and intentional self-inflicted injury, personality disorders, schizophrenia and other psychotic disorders, disruptive behavior disorders, and miscellaneous mental health disorders.

### Implementation of PDMP use mandates is not associated with changes in opioid prescribing, high-risk prescribing, or adverse events

Table 2 displays the difference-in-differences estimates of the association between implementation of PDMP use mandates and each outcome. Mandate implementation was not associated with changes in opioid prescribing (any discharge opioid prescription: −0.6% points, 95% CI: −1.8, 0.6; mean days supplied of discharge opioid prescription: 0.05 days, 95% CI: −0.2, 0.3; mean total MME of discharge opioid prescription: 9.0 MME, 95% CI: −4.7, 22.7; mean daily MME of discharge opioid prescription: 0.8 MME, 95% CI: −0.2, 1.9). Mandate implementation was also not associated with changes in high-risk opioid prescribing (days supplied > 7: −0.3% points, 95% CI: −2.3, 1.7; opioid-benzodiazepine overlap: −0.3% points, 95% CI: −0.8, 0.2) or with changes in the risk of an adverse event (−0.03% points, 95% CI: −1.0, 0.9).

**Table 2.** Association between implementation of PDMP use mandates and surgical outcomes among Medicare patients.

Outcome	Main analysis Coefficient (95% CI)	Sample size	Subgroup analysis (prior alcohol or substance use disorder) Coefficient (95% CI)	Sample size	Subgroup analysis (prior opioid use) Coefficient (95% CI)	Sample size	Subgroup analysis (states with strong mandates) Coefficient (95% CI)	Sample size
**Opioid prescribing**								
Probability of any discharge opioid prescription	−0.6 (−1.8, 0.6)	597 455	0.2 (−4.1, 4.4)	51 436	−0.3 (−2.6, 2.0)	255 286	−1.0 (−2.6, 0.6)	253 539
Days supplied of discharge opioid prescription	0.05 (−0.2, 0.3)	327 206	−0.6 (−1.6, 0.3)	23 540	0.3 (−0.09, 0.6)	119 214	0.02 (−0.3, 0.3)	142 416
Total MME of discharge opioid prescription	9.0 (−4.7, 22.7)	327 206	−26.8 (−75.8, 22.2)	23 540	19.7 (−0.7, 40.0)	119 214	9.3 (−9.4, 27.9)	142 416
Daily MME of discharge opioid prescription (MME/days supply)	0.8 (−0.2, 1.9)	327 206	1.4 (−1.6, 4.4)	23 540	0.9 (−0.3, 2.1)	119 214	1.0 (0.02, 2.1)	142 416
**High-risk opioid prescribing**								
Days supplied > 7	−0.3 (−2.3, 1.7)	327 206	−1.1 (−6.4, 4.2)	23 540	−0.3 (−2.8, 2.2)	119 214	−3.0 (−4.8, −1.1)	142 416
Indicator for opioid-benzodiazepine overlap	−0.3 (−0.8, 0.2)	327 206	−0.7 (−3.3, 1.8)	23 540	−0.2 (−1.2, 0.8)	119 214	0.2 (−0.2, 0.6)	142 416
**Adverse events**								
Any adverse event	−0.03 (−1.0, 0.9)	597 455	−1.6 (−5.0, 1.7)	51 436	−0.5 (−2.0, 1.0)	255 286	1.3 (−0.1, 2.8)	253 539

Source: The 2016-2019 Medicare claims data.

MME—morphine milligram equivalents; Opioid-benzodiazepine overlap for at least 1 day; The benzodiazepine could be dispensed within the 3-day window or before the index date—it does not matter, the point is whether there is any overlap at all. Adverse events outcome is composite for any of the following outcomes within 30 days of the index date: at least one claim with a diagnosis code for opioid overdose; at least one opioid-related ED visit or hospitalization; death; at least one emergency department visit; at least one hospitalization. The treatment group includes the following states: AK, AR, FL, HI, and TX.

**Figure 1.**
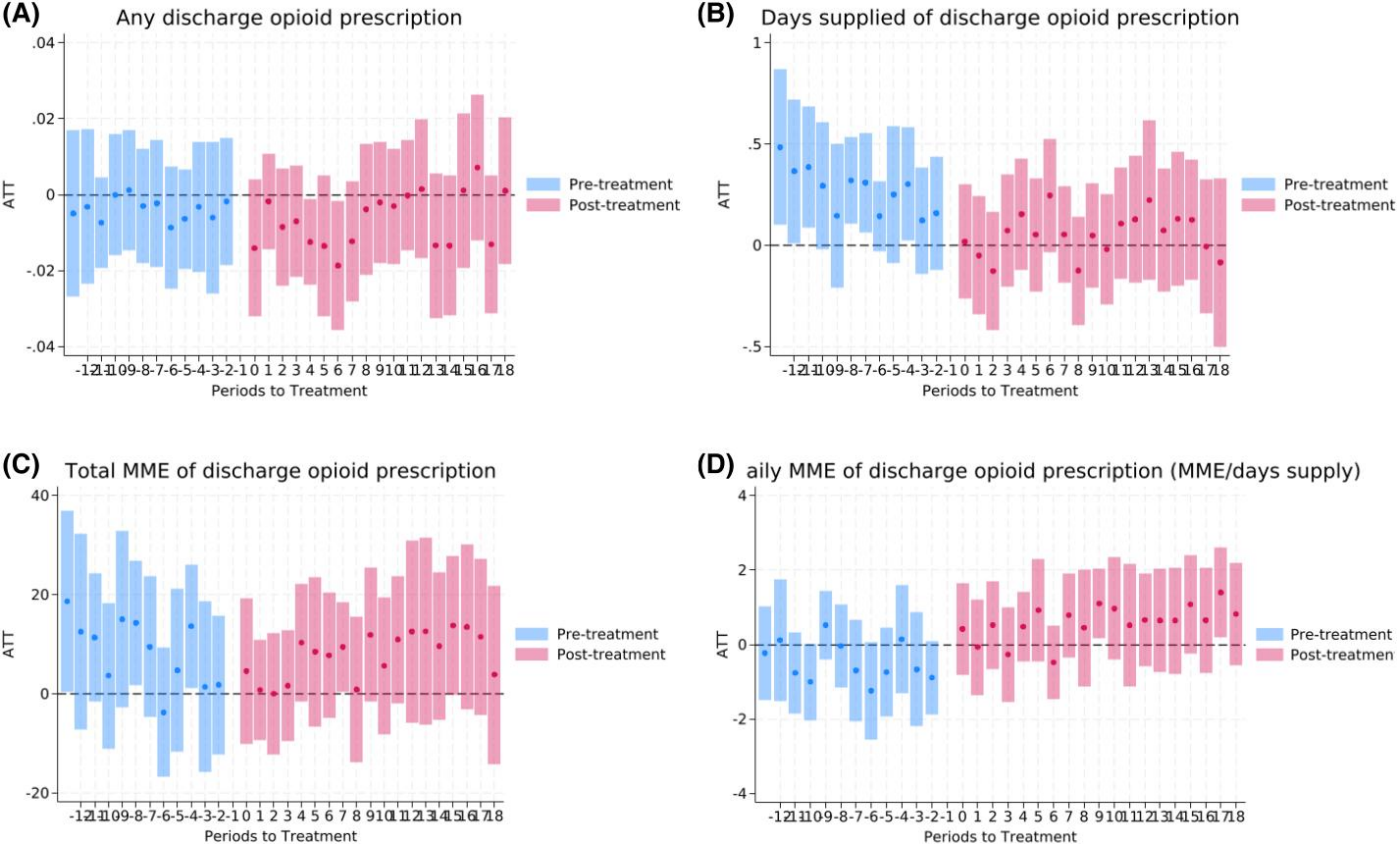
Event study plots for opioid prescribing outcomes. (A) Any discharge opioid prescription. (B) Days supplied of discharge opioid prescription. (C) Total MME of discharge opioid prescription. (D) Daily MME of discharge opioid prescription (MME/days supply). Note: ATT: Average Treatment Effect on the Treated, representing the estimated association of PDMP use mandates with opioid prescribing among patients in states that implemented the mandate relative to those in states that did not implement such mandates over time. Source: The 2016-2019 Medicare claims data.

**Figure 2.**
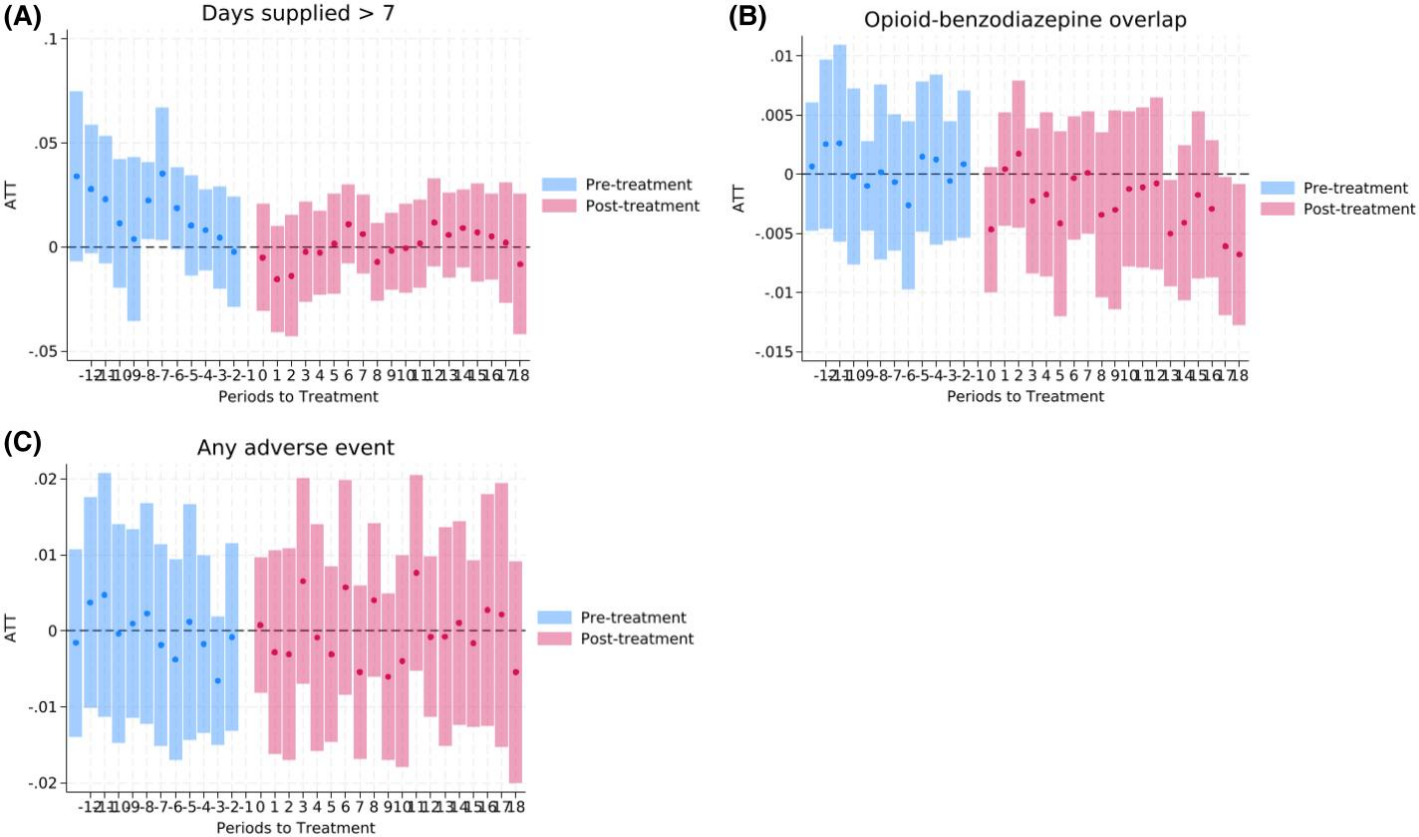
Event study plots for high-risk prescribing and adverse outcomes. (A) Days supplied >7. (B) Opioid-benzodiazepine overlap. (C) Any adverse event. Note: ATT: Average Treatment Effect on the Treated, representing the estimated association of PDMP use mandates with opioid prescribing among patients in states that implemented the mandate relative to those in states that did not implement such mandates over time. Source: The 2016-2019 Medicare claims data.

Figure 1 shows the event study estimates for each opioid prescribing outcome. Figure 2 shows the event study estimates for high-risk prescribing and adverse outcomes. None of the pre-intervention event study coefficients were statistically significant, except for a few related to supply days, total MME, and days supplied >7 outcomes. Similarly, none of the average pre-intervention coefficients were significant, except for days supplied. There was no evidence of dynamic effects over time during the post-intervention period.

### Implementation of PDMP use mandates is not associated with opioid-related outcomes among patients with substance use disorders or prior opioid use

Among patients with substance use disorders and those with prior opioid use, implementation of PDMP use mandates was not associated with changes in any outcome assessed, similar to the main analysis (Table 2). As shown in Appendix Figure S2 among patients with a history of substance use disorders, there were 1-2 significant pre-intervention event study coefficients for each outcome except for total MME; none of the pre-intervention event study average coefficients were significant. Similarly, Appendix Figure S3 shows 1-3 significant pre-intervention coefficients for days supplied, total MME, and adverse events outcomes, among patients with prior opioid use; none of the pre-intervention event study average coefficients were significant except for supply days.

### Stringent PDMP mandates show no major outcome changes aside from modest shifts in daily MME and prescriptions exceeding 7-day supply

Implementation of PDMP use mandates was also not associated with changes in any outcome assessed when limiting the treatment group to 5 states with the most stringent mandates except for an increase in mean daily MME of discharge opioid prescription (1.0 MME, 95% CI: 0.02, 2.1) and a decline in the likelihood of a discharge prescription exceeding a 7-day supply (−3.0% points, 95% CI: −4.8, −1.1) (Table 2). As shown in Appendix Figure S4, there were multiple significant pre-intervention event study coefficients for each outcome except for discharge prescription exceeding a 7-day supply. However, the means of the pre-intervention coefficients were not significantly different from zero except for overlapping discharge opioid-benzodiazepine prescriptions and adverse events.

In sensitivity analyses excluding states that implemented an opioid prescribing limit on the same date or within 1 month of a PDMP use mandate (Alaska, Colorado, Delaware, Florida, Maine, Michigan, Rhode Island, Washington, and West Virginia), findings were substantively unchanged (Appendix Table S5, Appendix Figure S5).

## Discussion

This study evaluated the association between implementation of PDMP use mandates during 2017-2018 and perioperative opioid prescribing, high-risk prescribing behaviors, and postoperative opioid-related adverse events among Medicare patients. There was no evidence of changes in any of these outcomes. Findings suggest that PDMP use mandates may be insufficient to achieve the intended goals of mitigating the harms of opioid prescribing in the perioperative setting.

Several studies have evaluated the association between PDMP use mandates and opioid prescribing and high-risk prescribing in the surgical context, with mixed results.^46-52^ Our study compares favorably to these studies given our use of national data, examination of mandates across multiple states relative to a control group, and use of a policy database developed by professional legal epidemiologists to obtain information on the effective dates and details of PDMP use mandates. In our study, even the strongest PDMP use mandates were not associated with changes in opioid prescribing, with the exception of a 1 MME increase in daily dosage (corresponding to one-fifth of a pill containing 5 mg hydrocodone), which is unlikely to represent significant changes in opioid prescribing behavior. These findings contrast with evidence from other settings, in which “must-access” PDMP use mandates have been associated with declines in opioid prescribing and high-risk prescribing.^25,26,39-41,43-45,49^ In addition to the differences in clinical settings, another potential explanation for the diverging findings is that prior studies on must-access PDMP use mandates typically focused on mandates implemented earlier than 2017-2018.

There are several potential reasons why implementation of PDMP use mandates was not associated with reductions in opioid prescribing or high-risk prescribing. One possibility is that adherence to the mandates was poor, perhaps because of the administrative burden of using these databases and concerns about stigmatizing patients.^45,78-81^. Another possibility is that the information provided in PDMPs on patients' prior controlled substance dispensing patterns is insufficient to meaningfully inform clinicians' opioid prescribing decisions following surgery. While PDMPs were designed in part to help clinicians identify dispensing patterns suggestive of misuse such as “doctor and pharmacy shopping,” the prevalence of such behaviors may be relatively low among surgical patients.

Our study adds to the limited evidence on the association between PDMP use mandates and the risk of adverse events in the surgical setting. The absence of a significant association in our findings stands in contrast to prior research demonstrating reductions in opioid-related inpatient stays and emergency department visits following PDMP mandates in broader, non-surgical contexts.^44^ Noteworthy, prior work focused on the Medicaid population, which differs from the surgical Medicare population examined here, potentially offering insight into the observed discrepancies.

This study's findings have policy and clinical implications. First, policymakers should consider coupling PDMP use mandates with other, more effective interventions to reduce high-risk opioid prescribing after surgery. These could include the development and implementation of procedure-specific opioid prescribing guidelines, mandatory clinician training on alternative pain management approaches, as well as educating patients on opioid-related risks and proper medication storage and disposal. Policymakers potentially could also pair PDMP use mandates with payer-specific opioid prescribing limits, some of which have been associated with reductions in surgical opioid prescribing without worsening pain control.^82-84^ Second, given that PDMP use mandates were not associated with changes in opioid prescribing, high-risk prescribing, or adverse events among Medicare patients with a history of a substance use disorder or prior opioid use—both risk factors for prescription opioid-related harms—policymakers and clinicians should implement other interventions to improve outcomes for these vulnerable populations, such as care coordination, individualized prescribing guidelines, naloxone co-prescribing, and counseling patients about safe opioid disposal practices.^85,86^

## Conclusion

Findings from this study suggest that implementation of PDMP use mandates was not associated with changes in perioperative opioid prescribing or the risk of adverse events after surgery. To achieve these goals, PDMP use mandates should be coupled with additional interventions, such as evidence-based opioid prescribing guidelines. Future research should explore the association between PDMP use mandates and opioid prescribing and opioid-related adverse events in other surgical populations, including Medicaid and commercially insured patients, and over longer follow-up periods. Additionally, given the wide variation in PDMP design features across states—registration requirements, query frequency, and enforcement mechanisms—future research should assess how these elements shape prescribing behavior and policy effectiveness to guide more targeted and safer PDMP implementation.

## Supplementary Material

qxaf218_Supplementary_Data
