# Supplementary material for: Prescription drug monitoring programs and perioperative opioid prescribing and adverse events
Source: Health Aff Sch. 2025 Nov 12;3(11):qxaf218. doi: 10.1093/haschl/qxaf218 (PMC12645282; doi:10.1093/haschl/qxaf218)

**Supplemental Materials**

**Table of Contents**

**Appendix Table 1.** Details on Prescription Drug Monitoring Programs (PDMP) use mandates

**Appendix Table 2.** State assignment into treatment and control groups

**Appendix Table 3.** List of opioids included and the used morphine milligram equivalents (MME) conversion factors

**Appendix Table 4.** Opioid overdose diagnosis codes

**eMethods 1.** Details on covariates

**Appendix Figure 1.** Sample inclusion and exclusion criteria

**Appendix Figure 2.** Event study plots for all outcomes – subgroup analysis (prior substance use disorder)

**Appendix Figure 3.** Event study plots for all outcomes – subgroup analysis (prior opioid use)

**Appendix Figure 4.** Event study plots for all outcomes – subgroup analysis (states with strong PDMP use mandates)

**Appendix Table 5.** Association between implementation of PDMP use mandates and surgical outcomes among Medicare patients, Sensitivity analysis

**Appendix Figure 5.** Event study plots for all outcomes – sensitivity analysis (excluding states with concurrent enactment of PDMP use mandates and opioid prescribing limits)

**Appendix Table 1.** Details on Prescription Drug Monitoring Programs (PDMP) use mandates

| **State** | **Effective date** | **Circumstances under which mandates are required before opioid prescribing** | **“Strong” mandate (i.e., requires PDMP query every time a surgeon prescribes a schedule II opioid)** |
| --- | --- | --- | --- |
| Alaska | 7/26/2017 | Every time for Schedule II-III opioids. | YES |
| Alabama | 3/9/17 | Opioid prescription with > 30 daily MME (MD must review at least twice per year). | NO |
| Arkansas | 8/1/2017 | Schedule II-III opioid (every time) | YES |
| Arizona | 10/1/2017 | Before initial opioid prescription and at least quarterly. | NO |
| California | 1/1/2017 | Schedule II-IV opioids - check before first time and at least every 4 months afterwards. | NO |
| Colorado | 5/21/2018 | PDMP use mandate only applies to second opioid prescriptions and exempts post-surgical pain lasting > 14 days. | NO |
| Florida | 7/1/2018 | For patients 16 years old and older, check every time for all opioid prescriptions | YES |
| Georgia | 7/1/2018 | Check before prescribing Schedule II opioids for the first time and every 90 days for continuing treatment assuming the prescription exceeds 3-day supply or 26 pills. | NO |
| Hawaii | 7/1/2018 | Before prescribing Schedule II-IV opioid, except in emergency situations for a supply of three days or less. | YES |
| Illinois | 1/1/2018 | Initial prescription for Schedule II opioids | NO |
| Maryland | 7/1/2018 | Initial opioid prescription exceeding three-day supply | NO |
| Maine | 1/1/2017 | Initial opioid prescription and every 90 days for continuing therapy | NO |
| Michigan | 6/1/2018 | Any opioid prescription exceeding a 3-day supply | NO |
| North Carolina | 6/25/2018 | Initial opioid prescription and every 3 months for continuing treatment | NO |
| North Dakota | 1/1/2018 | Any opioid prescription prescribed for over 12 weeks, every 6 months. | NO |
| New Hampshire | 1/1/2017 | Initial Schedule II-IV opioid, at least twice per year afterwards. | NO |
| South Carolina | 5/29/2017 | Prescription for Schedule II opioid exceeding five-day supply. If patient is established, then query every 3 months. | NO |
| Texas | 9/1/2017 | Before every opioid prescription | YES |
| Utah | 5/9/2017 | First time prescription for Schedule II-III opioid exceeding a three-day supply | NO |
| Wisconsin | 4/1/2017 | Every opioid prescription exceeding a three-day supply. | NO |
| Wyoming | 3/14/2018 | Initial II-V opioids, every three months thereafter. | NO |

**Appendix Table 2.** State assignment into treatment and control groups

| **Treatment states^⁋^** | | Alaska; Alabama; Arkansas; Arizona; California; Colorado; Florida; Georgia; Hawaii; Illinois; Maryland; Maine; Michigan; North Carolina; North Dakota; New Hampshire; South Carolina; Texas; Utah; Wisconsin; and Wyoming |
| --- | --- | --- |
| **Control states^†^** | | District of Columbia; Idaho; Kansas; Minnesota; Missouri; Mississippi; Montana; Nebraska; Oregon; and South Dakota |
| **Excluded states** | Enacted PDMP use mandates and opioid prescribing limits during 2017-2018 | Delaware; Rhode Island; Washington; and West Virginia |
|  | Enacted PDMP use mandates before 2020 but not during 2018-2019 | Connecticut; Indiana; Iowa; Kentucky; Louisiana; Massachusetts; New Jersey; New Mexico; Nevada; New York; Ohio; Oklahoma; Pennsylvania; Tennessee; Virginia; and Vermont |
| **^⁋^** Treatment states enacted Prescription Drug Monitoring Programs (PDMP) use mandates affecting perioperative opioid prescribing to adults in 2017-2018.  **^†^** Control states did not enact such PDMP use mandates during 2016-2019. | | |

**Appendix Table 3.** List of opioids included and the used morphine milligram equivalents (MME) conversion factors

| **Type of Opioid (strength units)** | **MME  Conversion Factor** |
| --- | --- |
| Butorphanol (mg) | 7 |
| Codeine (mg) | 0.15 |
| Dihydrocodeine (mg) | 0.25 |
| Fentanyl buccal or SL tablets, or lozenge/troche (mcg) | 0.13 |
| Fentanyl film or oral spray (mcg) | 0.18 |
| Fentanyl nasal spray (mcg) | 0.16 |
| Fentanyl patch (mcg) | 7.2 |
| Hydrocodone (mg) | 1 |
| Hydromorphone (mg) | 4 |
| Levomethadyl (mg/ml) | 8 |
| Levorphanol tartrate (mg) | 11 |
| Meperidine hydrochloride (mg) | 0.1 |
| Methadone (mg) | 3 |
| Morphine (mg) | 1 |
| Opium (mg) | 1 |
| Oxycodone (mg) | 1.5 |
| Oxymorphone (mg) | 3 |
| Pentazocine (mg) | 0.37 |
| Propoxyphene (mg) | 0.23 |
| Tapentadol (mg) | 0.4 |
| Tramadol (mg) | 0.1 |
| MME – morphine milligram equivalents. Source: CDC File of National Drug Codes for Opioid analgesics, and Linked Oral Morphine Milligram Equivalent Conversion Factors, 2020 Version. Atlanta, GA: Centers for Disease Control and Prevention; 2021. Available upon request at <https://www.cdc.gov/drugoverdose/resources/data.html> | |

**Appendix Table 4.** Opioid overdose diagnosis codes

| **Diagnosis code** | **description** |
| --- | --- |
| T400 | Poisoning by, adverse effect of and underdosing of opium |
| T400X | Poisoning by, adverse effect of and underdosing of opium |
| T400X1 | Poisoning by opium, accidental (unintentional) |
| T400X1A | Poisoning by opium, accidental (unintentional), initial encounter |
| T400X1D | Poisoning by opium, accidental (unintentional), subsequent encounter |
| T400X1S | Poisoning by opium, accidental (unintentional), sequela |
| T400X2 | Poisoning by opium, intentional self-harm |
| T400X2A | Poisoning by opium, intentional self-harm, initial encounter |
| T400X2D | Poisoning by opium, intentional self-harm, subsequent encounter |
| T400X2S | Poisoning by opium, intentional self-harm, sequela |
| T400X3 | Poisoning by opium, assault |
| T400X3A | Poisoning by opium, assault, initial encounter |
| T400X3D | Poisoning by opium, assault, subsequent encounter |
| T400X3S | Poisoning by opium, assault, sequela |
| T400X4 | Poisoning by opium, undetermined |
| T400X4A | Poisoning by opium, undetermined, initial encounter |
| T400X4D | Poisoning by opium, undetermined, subsequent encounter |
| T400X4S | Poisoning by opium, undetermined, sequela |
| T401 | Poisoning by and adverse effect of heroin |
| T401X | Poisoning by and adverse effect of heroin |
| T401X1 | Poisoning by heroin, accidental (unintentional) |
| T401X1A | Poisoning by heroin, accidental (unintentional), initial encounter |
| T401X1D | Poisoning by heroin, accidental (unintentional), subsequent encounter |
| T401X1S | Poisoning by heroin, accidental (unintentional), sequela |
| T401X2 | Poisoning by heroin, intentional self-harm |
| T401X2A | Poisoning by heroin, intentional self-harm, initial encounter |
| T401X2D | Poisoning by heroin, intentional self-harm, subsequent encounter |
| T401X2S | Poisoning by heroin, intentional self-harm, sequela |
| T401X3 | Poisoning by heroin, assault |
| T401X3A | Poisoning by heroin, assault, initial encounter |
| T401X3D | Poisoning by heroin, assault, subsequent encounter |
| T401X3S | Poisoning by heroin, assault, sequela |
| T401X4 | Poisoning by heroin, undetermined |
| T401X4A | Poisoning by heroin, undetermined, initial encounter |
| T401X4D | Poisoning by heroin, undetermined, subsequent encounter |
| T401X4S | Poisoning by heroin, undetermined, sequela |
| T402 | Poisoning by, adverse effect of and underdosing of other opioids |
| T402X | Poisoning by, adverse effect of and underdosing of other opioids |
| T402X1 | Poisoning by other opioids, accidental (unintentional) |
| T402X1A | Poisoning by other opioids, accidental (unintentional), initial encounter |
| T402X1D | Poisoning by other opioids, accidental (unintentional), subsequent encounter |
| T402X1S | Poisoning by other opioids, accidental (unintentional), sequela |
| T402X2 | Poisoning by other opioids, intentional self-harm |
| T402X2A | Poisoning by other opioids, intentional self-harm, initial encounter |
| T402X2D | Poisoning by other opioids, intentional self-harm, subsequent encounter |
| T402X2S | Poisoning by other opioids, intentional self-harm, sequela |
| T402X3 | Poisoning by other opioids, assault |
| T402X3A | Poisoning by other opioids, assault, initial encounter |
| T402X3D | Poisoning by other opioids, assault, subsequent encounter |
| T402X3S | Poisoning by other opioids, assault, sequela |
| T402X4 | Poisoning by other opioids, undetermined |
| T402X4A | Poisoning by other opioids, undetermined, initial encounter |
| T402X4D | Poisoning by other opioids, undetermined, subsequent encounter |
| T402X4S | Poisoning by other opioids, undetermined, sequela |
| T403 | Poisoning by, adverse effect of and underdosing of methadone |
| T403X | Poisoning by, adverse effect of and underdosing of methadone |
| T403X1 | Poisoning by methadone, accidental (unintentional) |
| T403X1A | Poisoning by methadone, accidental (unintentional), initial encounter |
| T403X1D | Poisoning by methadone, accidental (unintentional), subsequent encounter |
| T403X1S | Poisoning by methadone, accidental (unintentional), sequela |
| T403X2 | Poisoning by methadone, intentional self-harm |
| T403X2A | Poisoning by methadone, intentional self-harm, initial encounter |
| T403X2D | Poisoning by methadone, intentional self-harm, subsequent encounter |
| T403X2S | Poisoning by methadone, intentional self-harm, sequela |
| T403X3 | Poisoning by methadone, assault |
| T403X3A | Poisoning by methadone, assault, initial encounter |
| T403X3D | Poisoning by methadone, assault, subsequent encounter |
| T403X3S | Poisoning by methadone, assault, sequela |
| T403X4 | Poisoning by methadone, undetermined |
| T403X4A | Poisoning by methadone, undetermined, initial encounter |
| T403X4D | Poisoning by methadone, undetermined, subsequent encounter |
| T403X4S | Poisoning by methadone, undetermined, sequela |
| T404 | Poisoning by, adverse effect of and underdosing of other synthetic narcotics |
| T4041 | Poisoning by, adverse effect of and underdosing of fentanyl or fentanyl analogs |
| T40411 | Poisoning by fentanyl or fentanyl analogs, accidental (unintentional) |
| T40411A | Poisoning by fentanyl or fentanyl analogs, accidental (unintentional), initial encounter |
| T40411D | Poisoning by fentanyl or fentanyl analogs, accidental (unintentional), subsequent encounter |
| T40411S | Poisoning by fentanyl or fentanyl analogs, accidental (unintentional), sequela |
| T40412 | Poisoning by fentanyl or fentanyl analogs, intentional self-harm |
| T40412A | Poisoning by fentanyl or fentanyl analogs, intentional self-harm, initial encounter |
| T40412D | Poisoning by fentanyl or fentanyl analogs, intentional self-harm, subsequent encounter |
| T40412S | Poisoning by fentanyl or fentanyl analogs, intentional self-harm, sequela |
| T40413 | Poisoning by fentanyl or fentanyl analogs, assault |
| T40413A | Poisoning by fentanyl or fentanyl analogs, assault, initial encounter |
| T40413D | Poisoning by fentanyl or fentanyl analogs, assault, subsequent encounter |
| T40413S | Poisoning by fentanyl or fentanyl analogs, assault, sequela |
| T40414 | Poisoning by fentanyl or fentanyl analogs, undetermined |
| T40414A | Poisoning by fentanyl or fentanyl analogs, undetermined, initial encounter |
| T40414D | Poisoning by fentanyl or fentanyl analogs, undetermined, subsequent encounter |
| T40414S | Poisoning by fentanyl or fentanyl analogs, undetermined, sequela |
| T4042 | Poisoning by, adverse effect of and underdosing of tramadol |
| T40421 | Poisoning by tramadol, accidental (unintentional) |
| T40421A | Poisoning by tramadol, accidental (unintentional), initial encounter |
| T40421D | Poisoning by tramadol, accidental (unintentional), subsequent encounter |
| T40421S | Poisoning by tramadol, accidental (unintentional), sequela |
| T40422 | Poisoning by tramadol, intentional self-harm |
| T40422A | Poisoning by tramadol, intentional self-harm, initial encounter |
| T40422D | Poisoning by tramadol, intentional self-harm, subsequent encounter |
| T40422S | Poisoning by tramadol, intentional self-harm, sequela |
| T40423 | Poisoning by tramadol, assault |
| T40423A | Poisoning by tramadol, assault, initial encounter |
| T40423D | Poisoning by tramadol, assault, subsequent encounter |
| T40423S | Poisoning by tramadol, assault, sequela |
| T40424 | Poisoning by tramadol, undetermined |
| T40424A | Poisoning by tramadol, undetermined, initial encounter |
| T40424D | Poisoning by tramadol, undetermined, subsequent encounter |
| T40424S | Poisoning by tramadol, undetermined, sequela |
| T4049 | Poisoning by, adverse effect of and underdosing of other synthetic narcotics |
| T40491 | Poisoning by other synthetic narcotics, accidental (unintentional) |
| T40491A | Poisoning by other synthetic narcotics, accidental (unintentional), initial encounter |
| T40491D | Poisoning by other synthetic narcotics, accidental (unintentional), subsequent encounter |
| T40491S | Poisoning by other synthetic narcotics, accidental (unintentional), sequela |
| T40492 | Poisoning by other synthetic narcotics, intentional self-harm |
| T40492A | Poisoning by other synthetic narcotics, intentional self-harm, initial encounter |
| T40492D | Poisoning by other synthetic narcotics, intentional self-harm, subsequent encounter |
| T40492S | Poisoning by other synthetic narcotics, intentional self-harm, sequela |
| T40493 | Poisoning by other synthetic narcotics, assault |
| T40493A | Poisoning by other synthetic narcotics, assault, initial encounter |
| T40493D | Poisoning by other synthetic narcotics, assault, subsequent encounter |
| T40493S | Poisoning by other synthetic narcotics, assault, sequela |
| T40494 | Poisoning by other synthetic narcotics, undetermined |
| T40494A | Poisoning by other synthetic narcotics, undetermined, initial encounter |
| T40494D | Poisoning by other synthetic narcotics, undetermined, subsequent encounter |
| T40494S | Poisoning by other synthetic narcotics, undetermined, sequela |
| T404X | Poisoning by, adverse effect of and underdosing of other synthetic narcotics |
| T404X1 | Poisoning by other synthetic narcotics, accidental (unintentional) |
| T404X1A | Poisoning by other synthetic narcotics, accidental (unintentional), initial encounter |
| T404X1D | Poisoning by other synthetic narcotics, accidental (unintentional), subsequent encounter |
| T404X1S | Poisoning by other synthetic narcotics, accidental (unintentional), sequela |
| T404X2 | Poisoning by other synthetic narcotics, intentional self-harm |
| T404X2A | Poisoning by other synthetic narcotics, intentional self-harm, initial encounter |
| T404X2D | Poisoning by other synthetic narcotics, intentional self-harm, subsequent encounter |
| T404X2S | Poisoning by other synthetic narcotics, intentional self-harm, sequela |
| T404X3 | Poisoning by other synthetic narcotics, assault |
| T404X3A | Poisoning by other synthetic narcotics, assault, initial encounter |
| T404X3D | Poisoning by other synthetic narcotics, assault, subsequent encounter |
| T404X3S | Poisoning by other synthetic narcotics, assault, sequela |
| T404X4 | Poisoning by other synthetic narcotics, undetermined |
| T404X4A | Poisoning by other synthetic narcotics, undetermined, initial encounter |
| T404X4D | Poisoning by other synthetic narcotics, undetermined, subsequent encounter |
| T404X4S | Poisoning by other synthetic narcotics, undetermined, sequela |
| T406 | Poisoning by, adverse effect of and underdosing of other and unspecified narcotics |
| T4060 | Poisoning by, adverse effect of and underdosing of unspecified narcotics |
| T40601 | Poisoning by unspecified narcotics, accidental (unintentional) |
| T40601A | Poisoning by unspecified narcotics, accidental (unintentional), initial encounter |
| T40601D | Poisoning by unspecified narcotics, accidental (unintentional), subsequent encounter |
| T40601S | Poisoning by unspecified narcotics, accidental (unintentional), sequela |
| T40602 | Poisoning by unspecified narcotics, intentional self-harm |
| T40602A | Poisoning by unspecified narcotics, intentional self-harm, initial encounter |
| T40602D | Poisoning by unspecified narcotics, intentional self-harm, subsequent encounter |
| T40602S | Poisoning by unspecified narcotics, intentional self-harm, sequela |
| T40603 | Poisoning by unspecified narcotics, assault |
| T40603A | Poisoning by unspecified narcotics, assault, initial encounter |
| T40603D | Poisoning by unspecified narcotics, assault, subsequent encounter |
| T40603S | Poisoning by unspecified narcotics, assault, sequela |
| T40604 | Poisoning by unspecified narcotics, undetermined |
| T40604A | Poisoning by unspecified narcotics, undetermined, initial encounter |
| T40604D | Poisoning by unspecified narcotics, undetermined, subsequent encounter |
| T40604S | Poisoning by unspecified narcotics, undetermined, sequela |
| T4069 | Poisoning by, adverse effect of and underdosing of other narcotics |
| T40691 | Poisoning by other narcotics, accidental (unintentional) |
| T40691A | Poisoning by other narcotics, accidental (unintentional), initial encounter |
| T40691D | Poisoning by other narcotics, accidental (unintentional), subsequent encounter |
| T40691S | Poisoning by other narcotics, accidental (unintentional), sequela |
| T40692 | Poisoning by other narcotics, intentional self-harm |
| T40692A | Poisoning by other narcotics, intentional self-harm, initial encounter |
| T40692D | Poisoning by other narcotics, intentional self-harm, subsequent encounter |
| T40692S | Poisoning by other narcotics, intentional self-harm, sequela |
| T40693 | Poisoning by other narcotics, assault |
| T40693A | Poisoning by other narcotics, assault, initial encounter |
| T40693D | Poisoning by other narcotics, assault, subsequent encounter |
| T40693S | Poisoning by other narcotics, assault, sequela |
| T40694 | Poisoning by other narcotics, undetermined |
| T40694A | Poisoning by other narcotics, undetermined, initial encounter |
| T40694D | Poisoning by other narcotics, undetermined, subsequent encounter |
| T40694S | Poisoning by other narcotics, undetermined, sequela |

**eMethods 1.** Details on covariates

The model adjusts for conceptually relevant sociodemographic variables including patient’s age, sex, race/ethnicity, prior opioid use, any mental health disorder, alcohol or substance use disorder, Charlson co-morbidity index, surgeon’s specialty, as well as the type of procedure.

In this model, patient’s age was a continuous variable for patients 65 years old and older. Patient’s sex was a binary indicator for female and male patients. Patient’s race/ethnicity was a categorical variable using the RTI version in Medicare claims and consisted of the following categories: non-Hispanic White, non-Hispanic Black, non-Hispanic and other race, Asian-Pacific Islander, American Indian/Alaska Native, and Hispanic.

Prior opioid use was adjusted for as a binary indicator for any opioid dispensing in the 180 days prior to index date. Having any mental health disorder was a binary indicator for having any of the following disorders: adjustment disorders, anxiety disorders, mood disorders, suicide and intentional self-inflicted injury, personality disorders, schizophrenia and other psychotic disorders, attention-deficit conduct, disruptive behavior disorders, impulse control disorders, and other mental health disorders. Having any alcohol or substance use disorder was also adjusted for as a binary indicator. The Charlson co-morbidity index was a continuous variable taking the value 0 if the patient had no comorbid conditions, and a positive value between 1 and 21 depending on the presence of comorbidities.

Surgeon’s specialty was adjusted for as a categorical variable including 17 categories for different specialties: colon and rectal surgery; general surgery; hand surgery; neurological surgery; obstetrics and gynecology; orthopedic surgery; otolaryngology; pediatric surgery; plastic surgery; surgical critical care; surgical oncology; thoracic surgery (cardiothoracic vascular surgery); transplant surgery; trauma surgery; urology; vascular surgery; and multiple specialty. Surgeon’s specialty was retrieved from Medicare provider file.

Finally, the type of procedure was adjusted for as a categorical variable with 20 categories for the 20 most frequent procedures, one category for multiple procedures, and one category for everything else. The most frequent surgeries in descending order were: arthroplasty of knee; arthroscopy of knee; cholecystectomy – laparoscopic; laminectomy/corpectomy; arthroscopy of shoulder; nerve decompression; adjacent tissue transfer or rearrangement; arthroplasty of hip; hernia repair, inguinal/femoral – open; trigger finger release; transurethral resection of prostate; open treatment of fracture, upper extremity; arthrodesis of spine; hernia repair, inguinal/femoral – laparoscopic; endovascular revascularization, lower extremity; hernia repair, abdominal – open; arthroplasty of shoulder; thromboendarterectomy; skin graft; and arteriovenous fistula creation or reconstruction. The Table below shows the frequency of procedures by category in the total sample and by treatment status.

| **Procedure category** | **Total sample**  N=597,455  (100%) | **Treatment**  N=492,523  (82.4%) | **Control**  N=104,932  (17.6%) |
| --- | --- | --- | --- |
| Arthroplasty of knee | 36,976 (6.2%) | 28,740 (5.8%) | 8,236 (7.8%) |
| Arthroscopy of knee | 25,965 (4.3%) | 21,834 (4.4%) | 4,131 (3.9%) |
| Cholecystectomy – laparoscopic | 25,679 (4.3%) | 21,161 (4.3%) | 4,518 (4.3%) |
| Laminectomy/corpectomy | 23,097 (3.9%) | 18,805 (3.8%) | 4,292 (4.1%) |
| Nerve decompression | 21,711 (3.6%) | 17,348 (3.5%) | 4,363 (4.2%) |
| Arthroscopy of shoulder | 19,722 (3.3%) | 16,500 (3.4%) | 3,222 (3.1%) |
| Adjacent tissue transfer or rearrangement | 16,996 (2.8%) | 14,904 (3.0%) | 2,092 (2.0%) |
| Arthroplasty of hip | 15,430 (2.6%) | 11,708 (2.4%) | 3,722 (3.5%) |
| Hernia repair, inguinal/femoral – open | 14,528 (2.4%) | 12,190 (2.5%) | 2,338 (2.2%) |
| Trigger finger release | 13,330 (2.2%) | 11,130 (2.3%) | 2,200 (2.1%) |
| Transurethral resection of prostate | 12,555 (2.1%) | 10,567 (2.1%) | 1,988 (1.9%) |
| Open treatment of fracture, upper extremity | 9,751 (1.6%) | 8,174 (1.7%) | 1,577 (1.5%) |
| Endovascular revascularization, lower extremity | 9,589 (1.6%) | 8,155 (1.7%) | 1,434 (1.4%) |
| Arthrodesis of spine | 9,221 (1.5%) | 7,526 (1.5%) | 1,695 (1.6%) |
| Arthroplasty of shoulder | 9,161 (1.5%) | 7,111 (1.4%) | 2,050 (2.0%) |
| Hernia repair, abdominal – open | 8,829 (1.5%) | 7,318 (1.5%) | 1,511 (1.4%) |
| Hernia repair, inguinal/femoral – laparoscopic | 8,803 (1.5%) | 7,571 (1.5%) | 1,232 (1.2%) |
| Thromboendarterectomy | 8,287 (1.4%) | 6,784 (1.4%) | 1,503 (1.4%) |
| Arteriovenous fistula creation or reconstruction | 6,798 (1.1%) | 5,742 (1.2%) | 1,056 (1.0%) |
| Skin graft | 6,567 (1.1%) | 5,548 (1.1%) | 1,019 (1.0%) |
| Multiple procedures | 114,532 (19.2%) | 95,923 (19.5%) | 18,609 (17.7%) |
| Other procedures | 179,928 (30.1%) | 147,784 (30.0%) | 32,144 (30.6%) |

**Appendix Figure 1.** Sample inclusion and exclusion criteria

Number of procedures among Medicare beneficiaries in the years 2016 through 2020
(n=4,562,726)

Analytic sample (n=597,455)

Service provider was not a surgeon (n=258,182; 30.8%)

Sample (n=855,637)

Dual eligible (n=78,668; 8.4%)

Procedures with missing covariates (n=16,133; 1.7%)

Sample (n=934,305)

Sample (n=950, 438)

1. Procedures where there was another procedure in the 30-day look back or 30-day look-forward period (n=289,846; 18.9%)
2. Length of stay for any associated confinement was more than 30 days (n=15,600; 1.3%)
3. Patient was not discharged to home (n=277,087; 22.6%)

Sample (n= 1,532,971)

1. Invalid dosing data in opioid prescription (n=2; 0.0%)
2. Injectable opioid or benzo prescription fill from 180 days prior through 30 days after discharge (n=774; 0.05%)
3. Procedures in the top 1% and bottom 1% of the total MMEs among dispensed opioid prescriptions (n=6,981; 0.5%)

Sample (n=1,540,728)

Sample (n=1,859,825)

Age less than 65 (n=319,097; 17.2%)

No continuous enrollment in Medicare Part A, B and D from 180-day look-back through 30-day look-forward period (n=997,183; 34.9%)

Sample (n=2,857,008)

Not in the treatment or control states (n=1,705,718; 37.4%)

**Appendix Figure 2.** Event study plots for all outcomes – subgroup analysis (prior substance use disorder)


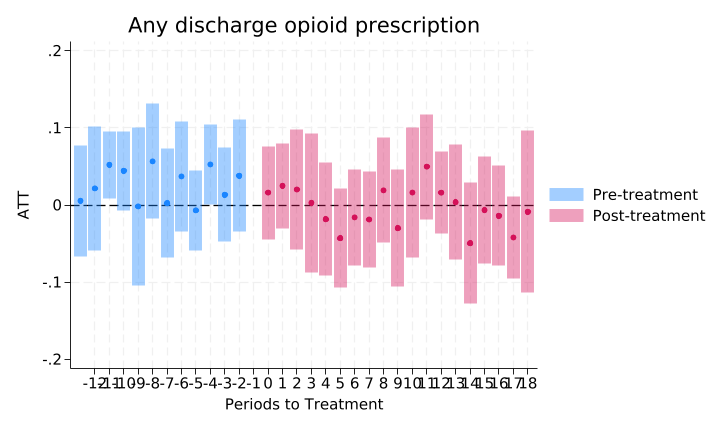

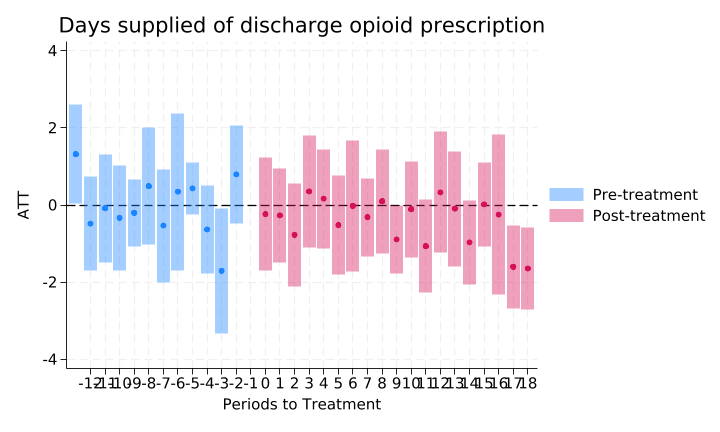

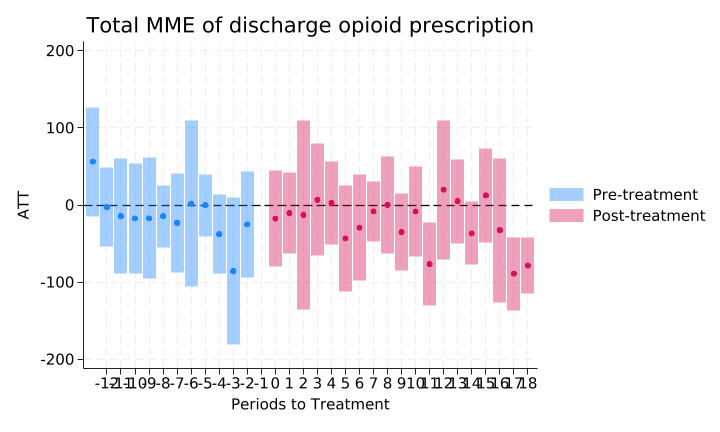

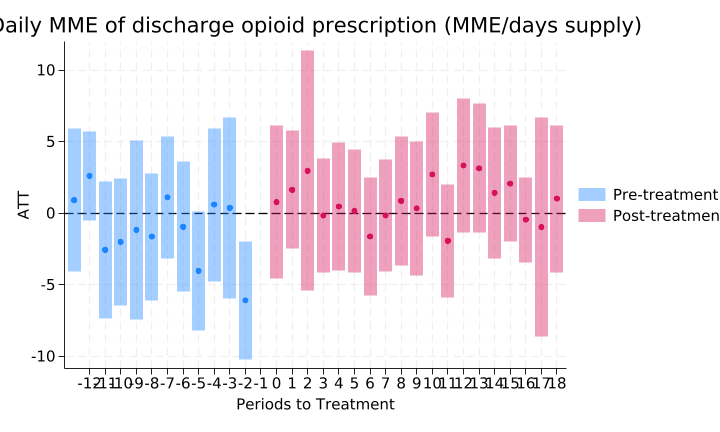

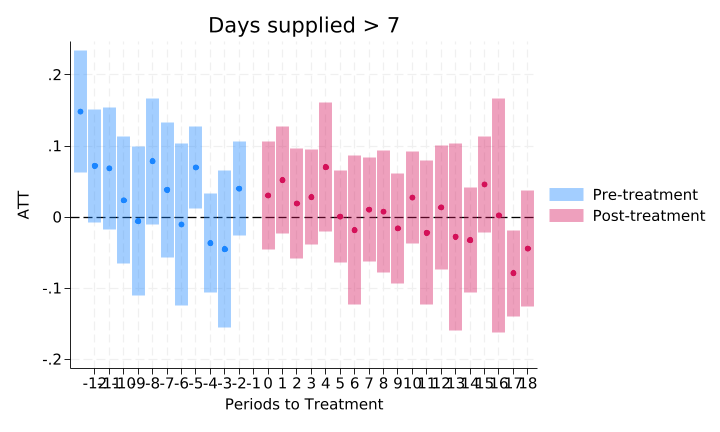

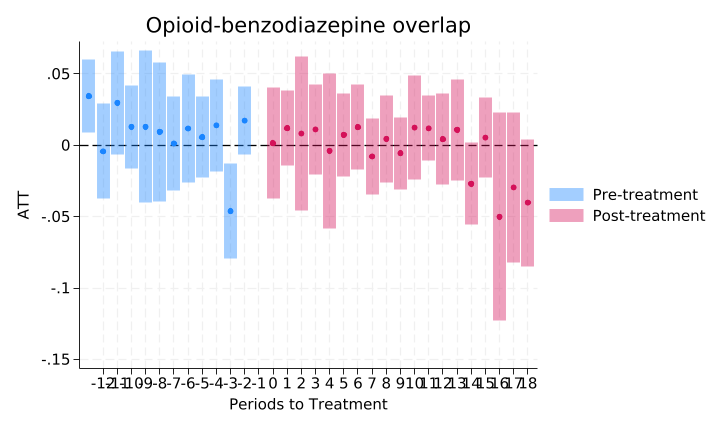


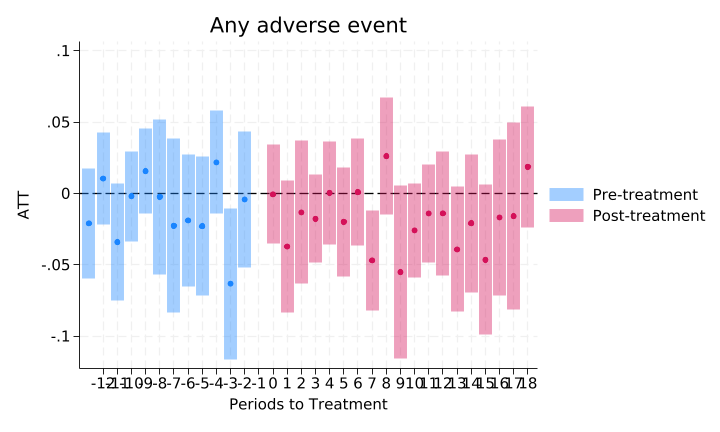


**Appendix Figure 3.** Event study plots for all outcomes – subgroup analysis (prior opioid use)

**
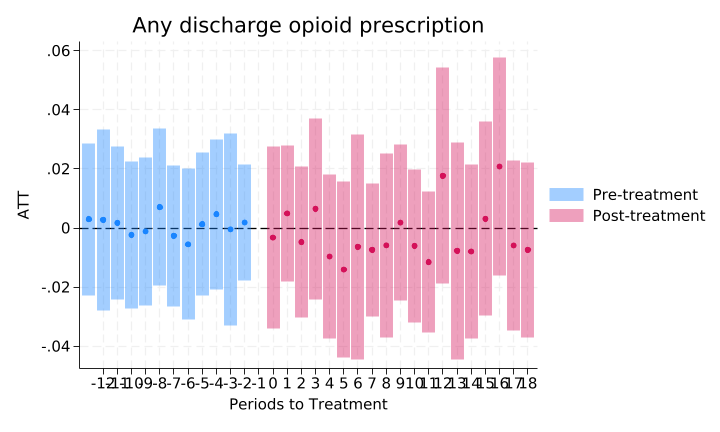

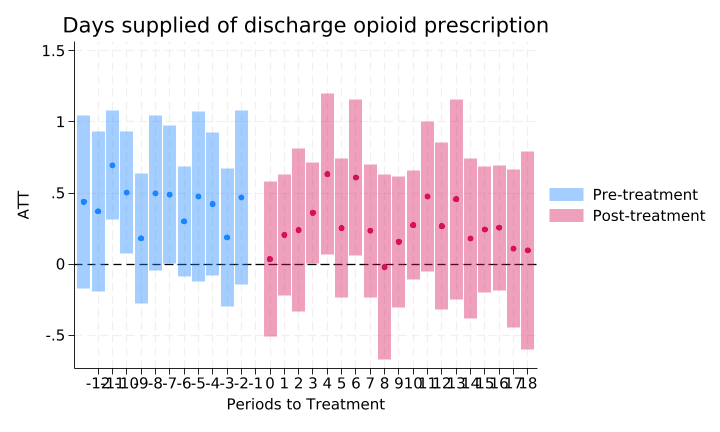

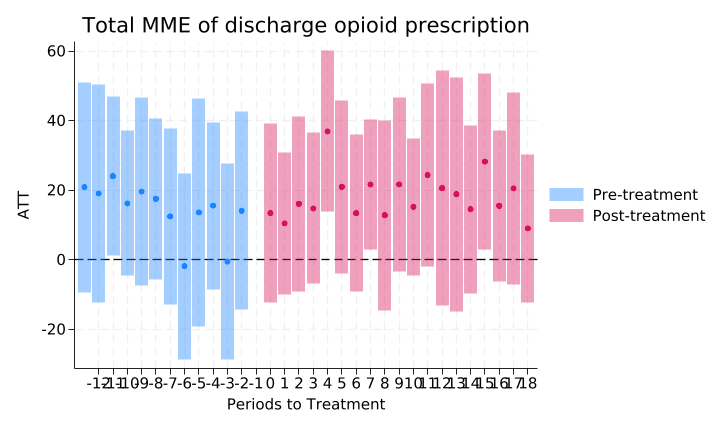

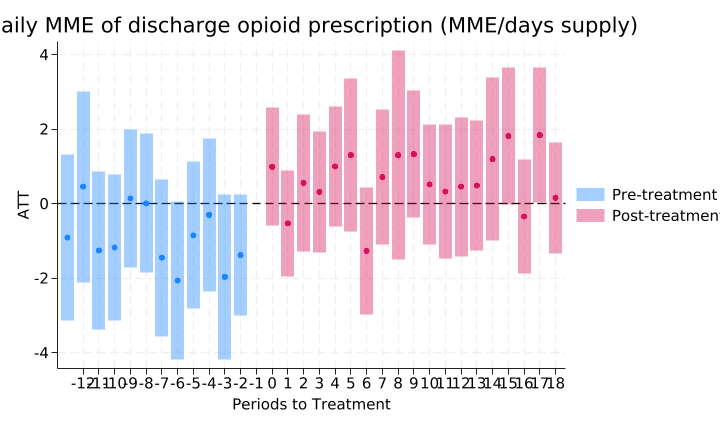

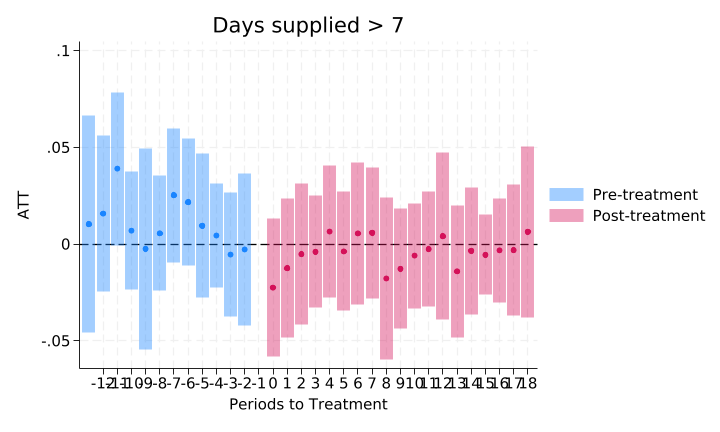

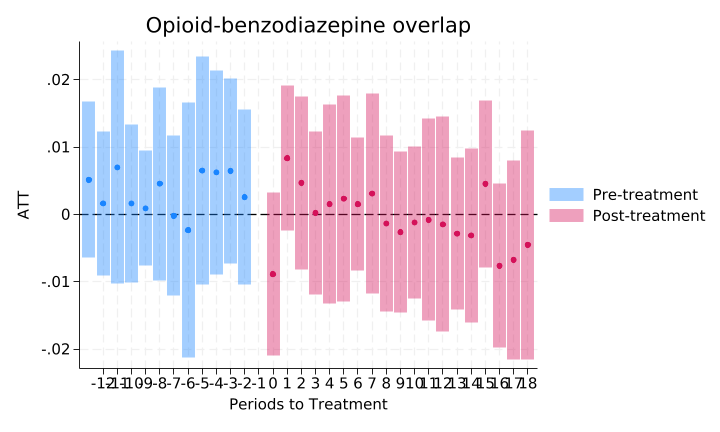
**

**
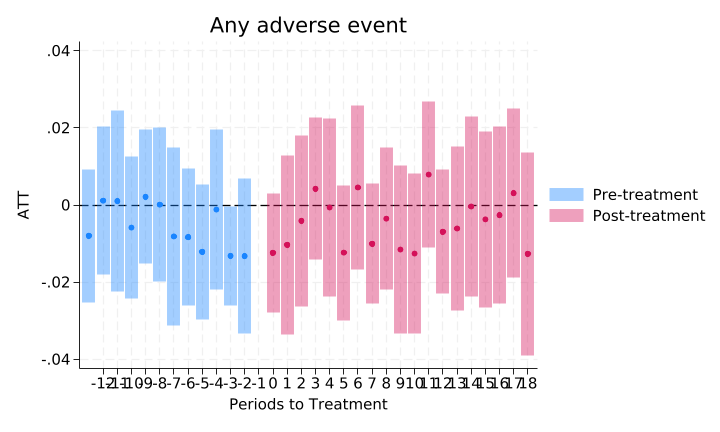
**

**Appendix Figure 4.** Event study plots for all outcomes – subgroup analysis (states with strong PDMP use mandates)


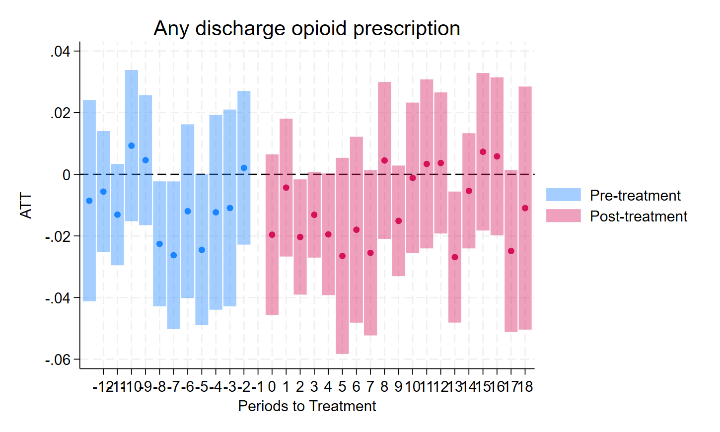

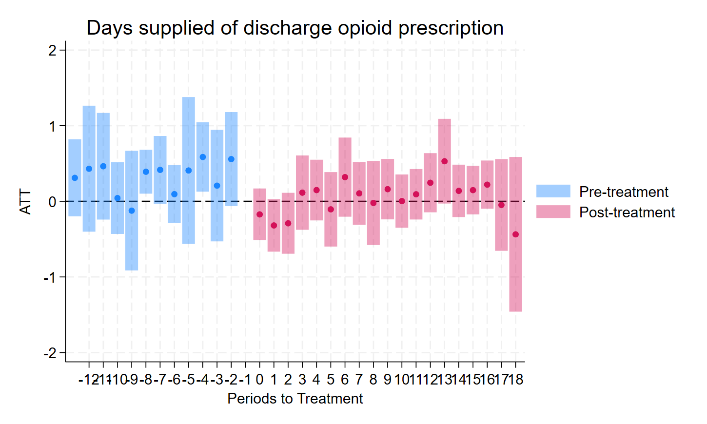


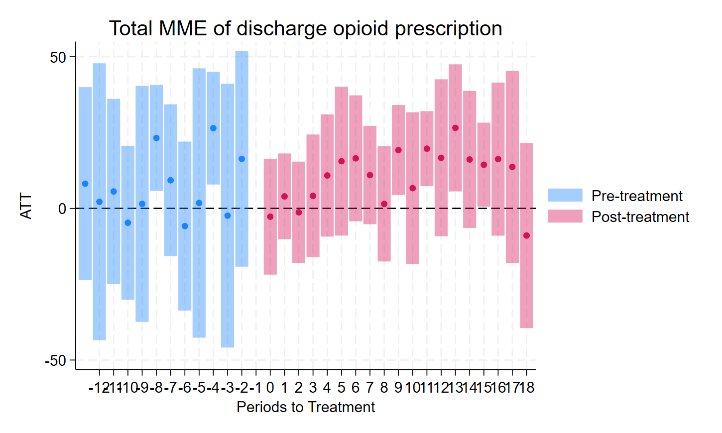

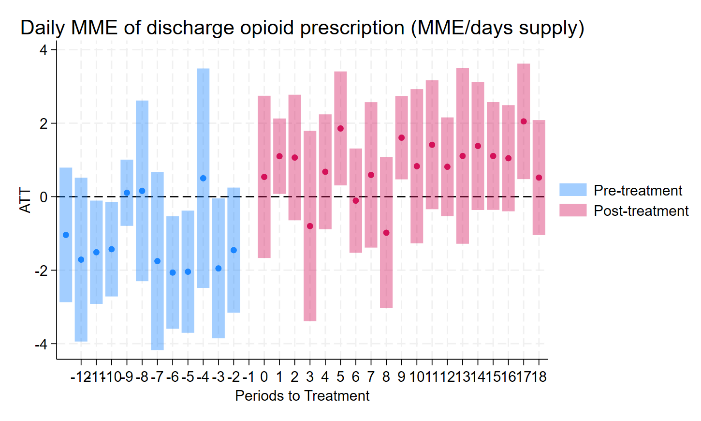

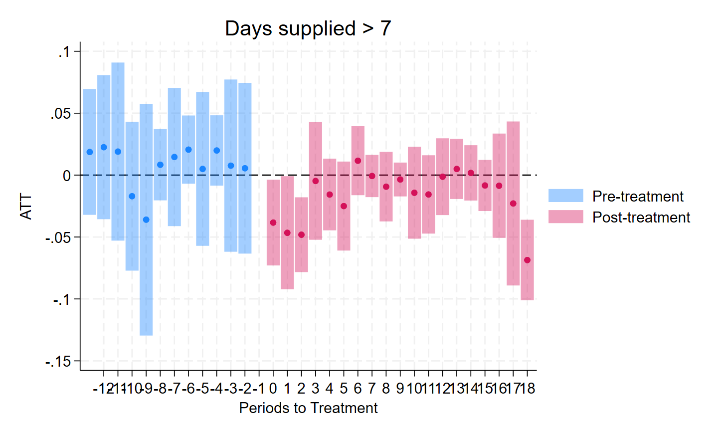

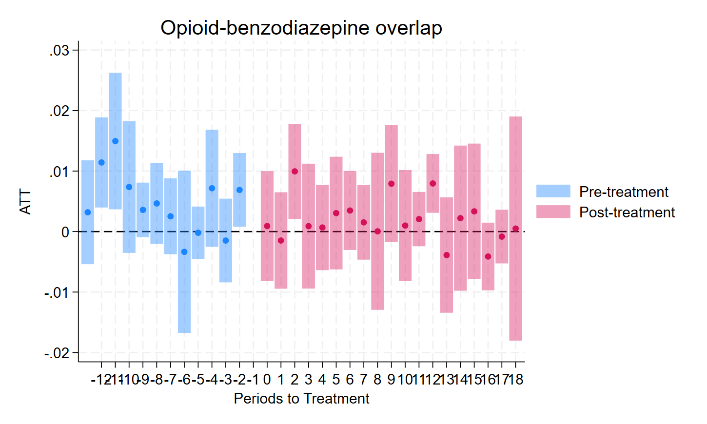

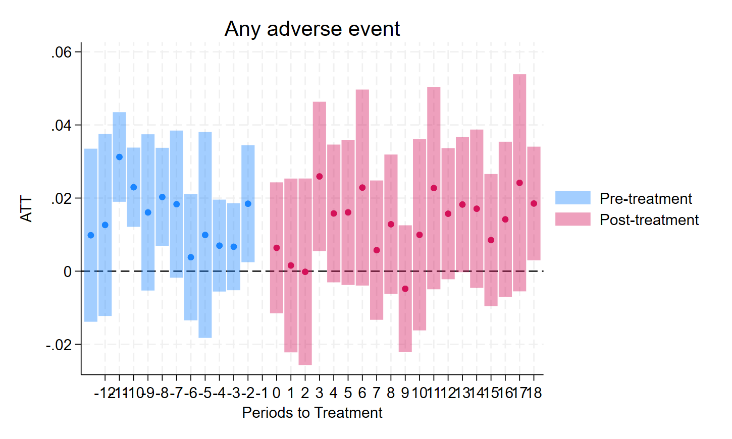


**Appendix Table 5.** Association between implementation of PDMP use mandates and surgical outcomes among Medicare patients, Sensitivity analysis

| **Outcome** | **Sensitivity analysis**  **Coefficient  (95% CI)** | **Sample size** |
| --- | --- | --- |
| **Opioid prescribing** | | |
| Probability of any discharge opioid prescription | -0.4  (-1.8, 0.9) | 460,678 |
| Days supplied of discharge opioid prescription | 0.09 (-0.2, 0.4) | 257,474 |
| Total MME of discharge opioid prescription | 10.1 (-9.1, 29.3) | 257,474 |
| Daily MME of discharge opioid prescription (MME/days supply) | 0.9 (-0.7, 2.5) | 257,474 |
| **High-risk opioid prescribing** | | |
| Days supplied > 7 | -0.3 (-3.1, 2.5) | 257,474 |
| Indicator for opioid-benzodiazepine overlap | -0.2 (-0.9, 0.5) | 257,474 |
| **Adverse events** | | |
| Any adverse event | -0.3 (-1.3, 0.7) | 460,678 |

**Appendix Figure 5.** Event study plots for all outcomes – sensitivity analysis (excluding states with concurrent enactment of PDMP use mandates and opioid prescribing limits)


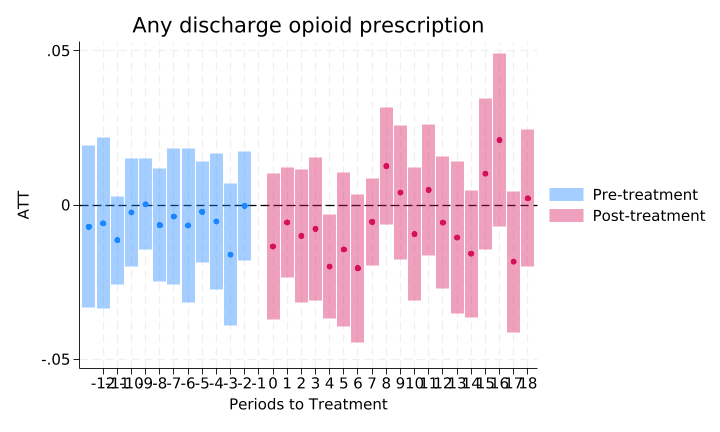

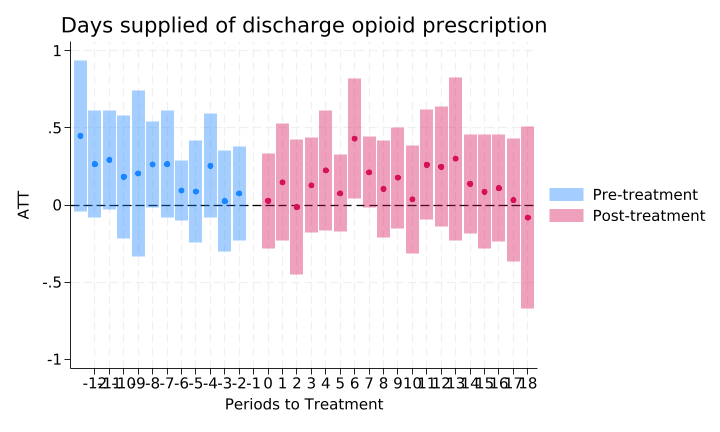


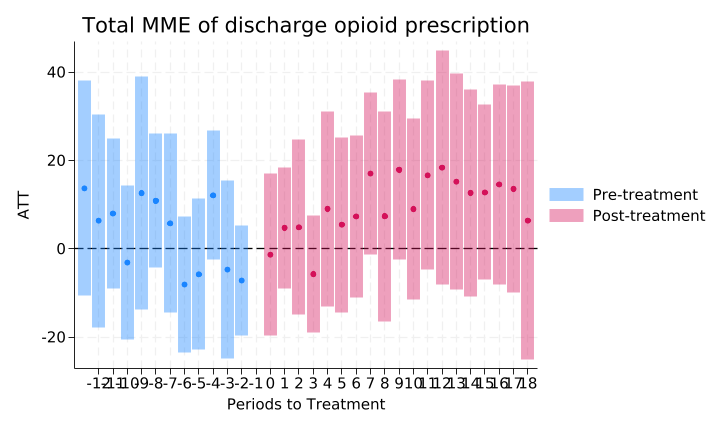

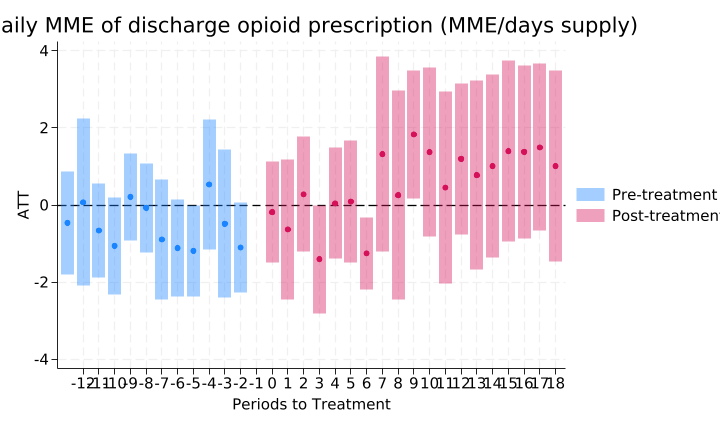


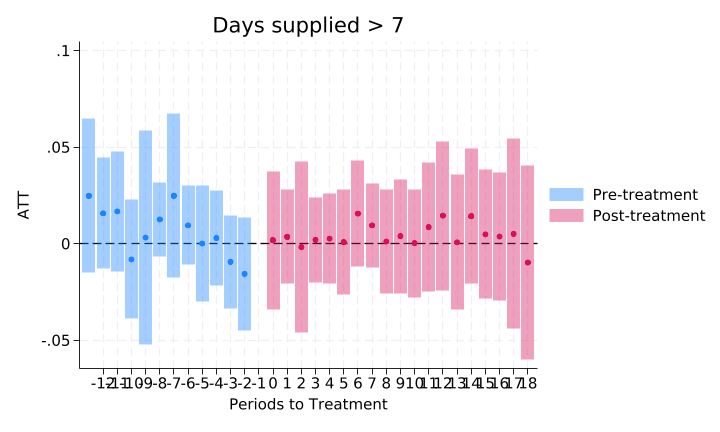

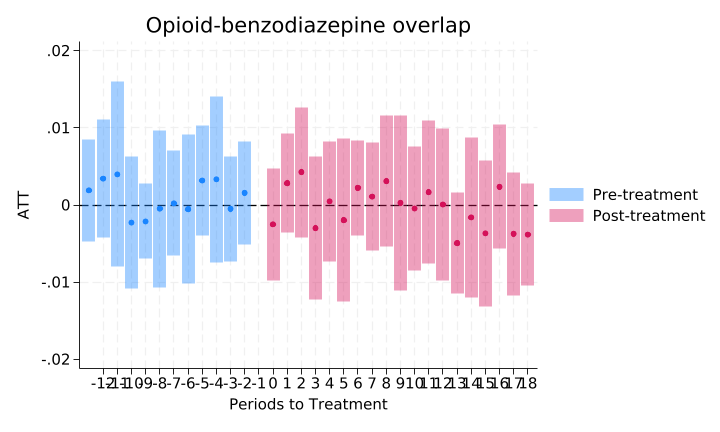


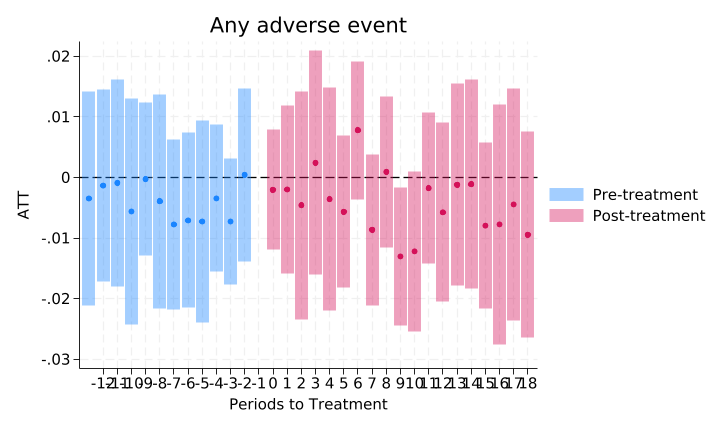

Supplement: qxaf218_Supplementary_Data [file qxaf218_supplementary_data.zip › 4. RR_OnlineSupplement_Clean_Version_updated.docx]
