# Supplementary material for: Prescription drug monitoring programs and perioperative opioid prescribing and adverse events
Source: Health Aff Sch. 2025 Nov 12;3(11):qxaf218. doi: 10.1093/haschl/qxaf218 (PMC12645282; doi:10.1093/haschl/qxaf218)
Supplement: qxaf218_Supplementary_Data [file qxaf218_supplementary_data.zip › 1. Authors Information.docx]

**Prescription Drug Monitoring Program Use Mandates and Perioperative Opioid Prescribing and Adverse Events**

**Authors:**

Joanne Constantin, PhD, MPH, Susan B. Meister Child Health Evaluation and Research Center, Department of Pediatrics, University of Michigan Medical School, Department of Health Policy and Management, School of Public Health, Department of Pediatrics, College of Medicine, State University of New York (SUNY) Downstate Health Sciences University, Brooklyn, NY, [Joanne.Constantin@downstate.edu](mailto:Joanne.Constantin@downstate.edu)

Jennifer Waljee, MD, MPH, Division of Plastic and Reconstructive Surgery, Indiana University School of Medicine, Indianapolis, IN, [jwaljee@iu.edu](mailto:jwaljee@iu.edu)

Thuy Nguyen, PhD, Department of Health Management and Policy, University of Michigan School of Public Health, Ann Arbor, MI, [thuydn@umich.edu](mailto:thuydn@umich.edu)

Amy Bohnert, PhD, Department of Anesthesiology, University of Michigan Medical School, Opioid Research Institute, Office of the Vice President for Research, University of Michigan, Ann Arbor, MI, [amybohne@med.umich.edu](mailto:amybohne@med.umich.edu)

Usha Nuliyalu, MPH, Susan B. Meister Child Health Evaluation and Research Center, Department of Pediatrics, University of Michigan Medical School, Ann Arbor, MI, [nuliusha@med.umich.edu](mailto:nuliusha@med.umich.edu)

Chad M. Brummett, MD, Department of Anesthesiology, University of Michigan Medical School, Opioid Research Institute, Office of the Vice President for Research, University of Michigan, Overdose Prevention Engagement Network, University of Michigan Medical School, Ann Arbor, MI, [cbrummet@med.umich.edu](mailto:cbrummet@med.umich.edu)

Kao-Ping Chua, MD, PhD, Department of Pediatrics, University of Michigan Medical School, Department of Health Management and Policy, University of Michigan School of Public Health, Ann Arbor, MI, [chuak@med.umich.edu](mailto:chuak@med.umich.edu)

**ACKNOWLEDGEMENTS**

Dr. Chua reports consulting fees from the U.S. Department of Justice for work outside the current manuscript. Dr. Brummett is a consultant for Vertex Pharmaceutical and Merck Pharmaceuticals, and he provides expert medicolegal consultation. Dr. Bohnert served as an expert witness for the State of Michigan in opioid-related litigation. No other disclosures were reported.

This study was supported by grant R01DA057284 from the National Institute on Drug Abuse (NIDA) to Dr. Chua and Dr. Waljee. Dr. Chua is also supported by NIDA grant R01DA056438. The funder played no role in the design and conduct of the study; collection, management, analysis, and interpretation of the data; preparation, review, or approval of the manuscript; and decision to submit the manuscript for publication.
